# Wet Paper Coding-Based Deep Neural Network Watermarking

**DOI:** 10.3390/s22093489

**Published:** 2022-05-04

**Authors:** Xuan Wang, Yuliang Lu, Xuehu Yan, Long Yu

**Affiliations:** 1 College of Electronic Engineering, National University of Defense Technology, Hefei 230037, China; wangxuan21d@nudt.edu.cn (X.W.); yanxh17@nudt.edu.cn (X.Y.); yl@nudt.edu.cn (L.Y.); 2 Anhui Province Key Laboratory of Cyberspace Security Situation Awareness and Evaluation, Hefei 230037, China

**Keywords:** deep neural network, watermarking, wet paper encoding, embedding rate

## Abstract

In recent years, the wide application of deep neural network models has brought serious risks of intellectual property rights infringement. Embedding a watermark in a network model is an effective solution to protect intellectual property rights. Although researchers have proposed schemes to add watermarks to models, they cannot prevent attackers from adding and overwriting original information, and embedding rates cannot be quantified. Therefore, aiming at these problems, this paper designs a high embedding rate and tamper-proof watermarking scheme. We employ wet paper coding (WPC), in which important parameters are regarded as wet blocks and the remaining unimportant parameters are regarded as dry blocks in the model. To obtain the important parameters more easily, we propose an optimized probabilistic selection strategy (OPSS). OPSS defines the unimportant-level function and sets the importance threshold to select the important parameter positions and to ensure that the original function is not affected after the model parameters are changed. We regard important parameters as an unmodifiable part, and only modify the part that includes the unimportant parameters. We selected the MNIST, CIFAR-10, and ImageNet datasets to test the performance of the model after adding a watermark and to analyze the fidelity, robustness, embedding rate, and comparison schemes of the model. Our experiment shows that the proposed scheme has high fidelity and strong robustness along with a high embedding rate and the ability to prevent malicious tampering.

## 1. Introduction

With the wide application of artificial intelligence technology, the applications represented by deep neural network (DNN) models are becoming increasingly extensive. A DNN is a computational model that mimics the structure and function of a biological central nervous system, and can be used to estimate complex functions [1]. It is calculated by connecting a limited number of neurons, with the function of each neuron being to calculate the result of the nonlinear mapping of a weighted vector. A DNN is a system that can learn and generalize; it can mine rules from known data to make inferences and decisions about unknown data. Because a deep representation learning algorithm based on a DNN won the ImageNet International Computer Vision Competition in 2012, DNNs have received extensive attention and research in both academia and industry, and have achieved great success in many fields of application. It is not difficult to foresee that neural networks will play an increasingly important role in serving different aspects of human society. However, as a digital product, while a neural network model can condense the designers’ wisdom, it requires considerable training data and computing resources. For example, in order to accurately recognize faces, tens of millions of face images are usually required for neural networks to learn and generalize. In addition, neural networks are affected by their network structure, data scale, and computing resources, with results often taking up to several weeks to calculate. Due to the very large cost of building a well-trained neural network model such models have become an important asset, and many manufacturers provide corresponding services. Users only need to remotely access an application programming interface (API) in order to easily use a relevant model for learning [2]. Moreover, the malicious use or illegal dissemination of neural network models prompts an urgent need for copyright protection; neural network watermarking can effectively solve this problem. Therefore, it is necessary to protect the intellectual property rights of neural network models from infringement.

**Figure 1:**
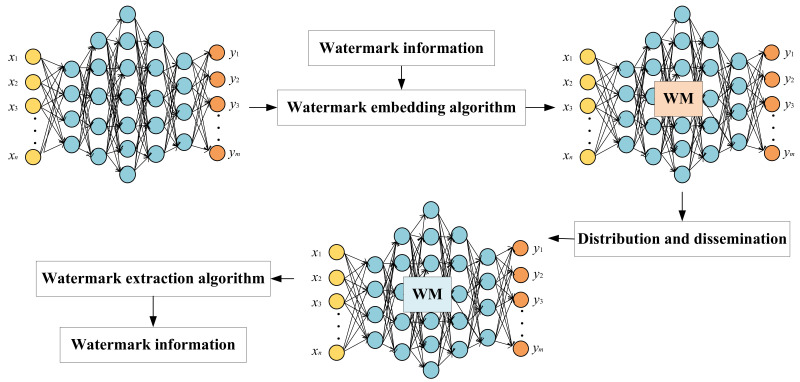
Neural network watermarking framework.

The basic framework of neural network watermarking is shown in Figure 1. In the embedding process, watermark information is embedded into a network through a watermark embedding algorithm. After the watermark embedding model is distributed and applied by a contractor, a watermark extraction algorithm is used to extract the watermark information from the model.

Recently, several researchers have proposed embedding digital watermarks in neural network models to protect developers’ intellectual property. Uchida et al. [3] first proposed a digital watermarking scheme; the core idea was to embed watermark information consisting of strings of bits in a regularized form into the weight distribution of one of the middle layers. Fan et al. [4] proposed adding a passport layer to neural networks for intellectual property rights protection. A new watermark embedding scheme with a compensation mechanism was proposed by Feng and Zhang [5]. Zhang et al. [6] used a spatial invisible watermarking mechanism, and proposed the first model watermarking framework for protecting image processing models. Different from the above literature, in which watermarks were embedded into the static content of models, Rouhani et al. [7] proposed embedding strings into probability density functions (PDFs) of different network layers; in this way, the watermark information is embedded in the dynamic content of a DNN. Generating a watermark depends on both the data and the model. The above schemes belong to the white box model category [8]. In the verification process of these schemes, the owner needs to know the internal details of the suspicious model (such as the structure and parameters) in order to extract its complete watermark and compare the bit error with the embedded watermark to complete the verification. Therefore, researchers have proposed methods for adding watermarks to black box models [8] to permit the watermark to be verified without needing to know the details, for instance, the model parameters. A black box model is a watermarking method that applies backdoor technology to ownership protection. In the process of model training or fine-tuning, the model owner adds an abstract sample (with a different distribution from the training sample) as a backdoor sample to the model training set in order to ensure the accuracy of the model in the original task. The abstract sample is used as the backdoor watermark for ownership authentication and can be triggered by passing the specific input data to the model, making the watermark more flexible and difficult to detect. Zhong et al. [9] proposed a method of adding labels for key samples and attempted to design a watermark that does not distort the original decision boundary. Considering that model watermarks may not have strong robustness to pruning with fine-tuning, Namba R et al. [10] proposed an exponentially weighted backdoor watermark method, which applies the influence of a backdoor watermark on parameters to the larger weight parameters (exponentially weighted implementation) in order to ensure that the watermark is more robust in pruning with fine-tuning. Similarly, in order to increase robustness against distillation attacks, a watermark scheme proposed by Szyller S et al. [11] was deployed in the prediction API of a model; the watermark is dynamically added to queries by changing the prediction response of the client. Researchers with Tencent Suzaku Lab [12] proposed a method for hiding malware in a neural network model that is similar to using least-significant-bit (LSB) image implications [13] by modifying the last few model parameters according to malicious code in order to guarantee that it does not affect the original model’s performance. Wang et al. [14] proposed a malware embedding method of neuronal parameters based on a neural network model and decomposed the binary bits of malware sequentially to embed the neuron parameters. Each parameter has 32-bit floating point numbers, and the first 8 bits remain unchanged; in the latter 24-bit embedding secret information, the embedded capacity is increased.

The above methods involve adding additional information into a DNN. The embedded watermark information can prove the ownership of a model, and embedded malicious code can be used for model attacks. Although the above schemes maintain high robustness against watermark removal attacks, there are several unresolved problems. First, the embedded information of the model is not quantified, and all methods require a great deal of space to embed information; second, they cannot be used to solve the fraudulent ownership claims of an adversary. That is, if an adversary forges additional watermarks for a model through an ambiguity attack, both the original and new watermarks can be detected by the verification program, and the ownership of the model cannot be determined. The method presented here can prevent the addition of information, meaning that a watermark cannot be tampered with, and the proposed scheme quantifies the embedded information of a model.

For the existing embedding watermark models, the embedding position of a watermark is not flexible and random. Moreover, due to the limited embedding capacity an embedded watermark is easily detected, and an attacker who has tampered with it can easily deny the veracity of the original. The proposed optimized probabilistic selection strategy (OPSS) computes the importance of convolution layer neuron parameters, defines the unimportant-level function, and sets the importance threshold to ensure that the parameter change does not affect most of the characteristics of the model. It can make full use of redundant neurons and increase the embedding rate in order to solve the problem of insufficient embedding rate mentioned above. In wet paper coding (WPC), a dry block has the characteristics of transmitting information, while a wet block cannot be used for this purpose. We select the important parameters as wet blocks and the rest of the parameters as dry blocks for watermark steganography. In the process of steganography, dry blocks are randomly selected for steganography, and an attacker can not accurately locate the location of steganography, which means that the watermark created using this method cannot be tampered with. Thus, the contributions of this article are as follows:

1. Using the proposed OPSS, the watermark’s embedding position can be selected. The OPSS includes two selection operations; Selection 1 is a level selection operation for filtering out important convolution layers, while Selection 2 sublimates the selected results in Selection 1 to obtain the important parameter positions of unimportant layers. This double selection strategy ensures that the important layers and important parameters are not destroyed and provides randomness and security guarantees for the subsequent steganography, which is not easy to detect.
2. By skillfully employing WPC technology, we take the selected important parameters of the model as the wet block and the rest as the dry block, and embed the secret information into the dry block. This embedding method increases the embedding capacity as much as possible and makes the model more stable.
3. Compared with the current watermarking models, this scheme makes steganography more random. The steganography capacity can be increased as much as possible while ensuring the normal operation of the model, and the attacker cannot deny the original watermark by tampering with it due to the large amount of embedded information.

## 2. Preliminaries

In this section, we introduce a number of previous studies as the basis for our proposed scheme. First, we introduce a probabilistic selection strategy (PSS) for selecting the important parameters of the model. Second, we introduce the principle of WPC technology, which enables a watermark to be added to the modifiable part selected in the previous step. Table 1 shows a list of all the parameters used in the paper.

### 2.1. PSS

In order to achieve better model performance, neural networks have recently been developed to be deeper. For example, VGG19 [15] has 16 convolutional layers. By deepening the network structure, a network is able to extract more abstract high-order features; for example, a 512-channel feature map has finally been extracted. Each layer has a large number of parameters, and there is redundant space. RESNet152 [16], for instance, has 151 convolutional layers. In order to improve the performance of a model, the depth of the model is deepened, resulting in more convolution layers. It is obvious that there are many convolution layers in these models. However, if each neural network parameter is to be changed, the calculation costs will be very large. Several studies [17,18,19,20] have shown that the parameters in a CNN model are not equally important, and certain parameters contain more useful information for a given task. Intuitively, the importance of neural network parameters can be measured by the degree of degradation in a model’s performance caused by removing certain parameters. However, the neurons of a convolutional neural network respond differently to different inputs, meaning that the importance of the parameters is input-dependent. After a sample is fed into a network, there exists a parameter set that makes the model’s performance decrease the fastest when it is removed. To obtain the importance of each parameter, it is possible to consider feeding all of the samples and then count the frequency at which each parameter is selected as an indication of parameter importance.

Tian et al. [21] proposed a PSS model to determine the importance of parameters in a CNN model and found that encrypting less than 8% of the convolutional layer parameters can effectively reduce the performance of the model. PSS uses the greedy search idea to construct a discrete optimization problem for model parameter selection, uses reparameterization technology to serialize the problem, and finally uses the automatic derivation framework to obtain the importance of each parameter. The authors identified the importance of parameters by evaluating the performance degradation of the pretrained model without these parameters. However, the neurons in a CNN model may have different responses for various inputs, implying that the importance of parameters is related to the inputs.

Therefore, the important parameters, $\hat{\Theta^{l}}$, are selected from the convolutional layers of $F_{\Theta }$. When feeding a sample $x_{n}=\left(n=1,2,\ldots ,N\right)$ into $F_{\Theta }$, there exists a parameter subset $\hat{\Theta^{l}}$ of $\Theta^{l}$ that causes the maximal performance degradation of $F_{\Theta }$ when $\hat{\Theta^{l}}$ is removed. Clearly, $\hat{\Theta^{l}}$ changes with the input, $x_{n}$. In order to eliminate such randomness, the number of times each parameter θ in $\Theta^{l}$ is selected as a candidate of $\hat{\Theta^{l}}$ after feeding all $x_{n}$ is counted. The selected frequency of a parameter θ in $\Theta^{l}$ is denoted by $p_{\theta }$. It is clear that the frequency, $p_{\theta }$, directly reflects the importance of a parameter θ to the pretrained model. Thus, $p_{\theta }$ is the importance of a parameter θ. For simplicity, $\hat{\Theta^{l}}$ dominates the set of the *l*-th layer and the parameters in it are called dominated parameters. Naturally, $\hat{\Theta }$ is the dominated set of $F_{\Theta }$.

Finally, the PSS approach is formulated in the following optimization problem, Equation (1),

(1) $$\min_{p_{\theta }\in \left[0,1\right]}\frac{1}{N}\sum_{n=1}^{N}L(F\left(x_{n},\left(I-Z^{\left(n\right)}⊙\Theta^{l}\right),y_{n}\right)+\lambda \|Z^{\left(n\right)}{\|}_{0}$$

where $Z^{\left(n\right)}={\left\{z_{\theta }^{\left(n\right)}\right\}}_{\theta \in \Theta^{l}},z_{\theta }^{\left(n\right)}∼Bern\left(z_{\theta }|p_{\theta }\right)$ is a sample of the binary random variable $z_{\theta }$, $\Theta^{l}$ denotes the parameters of the *l*-th convolutional layer, *I* is a vector of ones with the same length as $\Theta^{l}$, and λ is a weighting factor for the regularization term. The elementwise multiplication $\left(I-Z^{\left(n\right)}\right)⊙\Theta^{l}$ is designed to simulate the removal operation by noting that a parameter θ will be removed from $\Theta^{l}$ if the corresponding $z_{\theta }^{\left(n\right)}=1$. Thus, the first term indicates the performance of $F_{\Theta }$ after removing a part of the parameters (L(…) is a performance evaluation function). In order to maximize the performance degradation of $F_{\Theta }$, which is equivalent to minimizing the first term in (1), the important parameters θ in $\Theta^{l}$ should be assigned a large importance ($p_{\theta }$) such that $z_{\theta }^{\left(n\right)}=1$ for most ${x_{n}}^{’}$s. Thus, we can determine the importance ($p_{\theta }$) of each θ in $\Theta^{l}$ by solving the problem. It should be noted that the term $\|Z^{\left(n\right)}{\|}_{0}$ penalizes the number of removed parameters such that fewer parameters are assigned large importance values.

**Figure 2:**
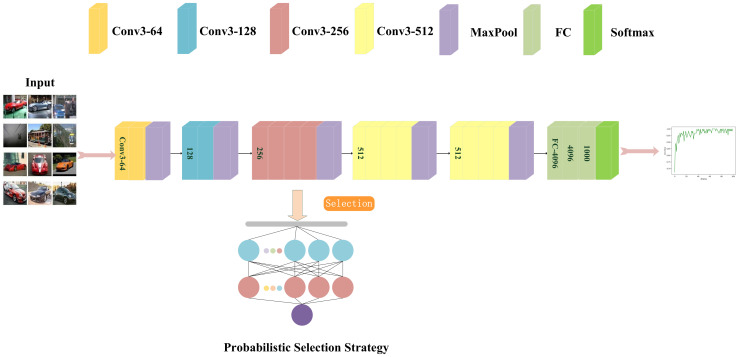
A schematic diagram of the PSS.

Figure 2 shows a schematic diagram of the selection process of the PSS. Several parameters are introduced above and used later. The above is a PSS model for the importance of neuron parameters in the calculation model. This paper proposes an OPSS which takes into account the hierarchical importance and calculation cost, which is described in detail in Section 3.

### 2.2. WPC

This paper proposes using WPC techniques to embed model watermarks. Wet paper refers to a portion of a paper that has been moisturized by water and cannot be used for writing. In this scenario, the important parameters are referred to as a wet block, and the other parameters are used for information transmission. A recipient can only read the information, and it is not known whether a parameter has been changed; hence, the safety of the model watermark is guaranteed from the sender side. The amount of valid transmission information in the storage information can be approximately equal to the number of normal storage units, which can be referred to as the number of unimportant parameters.

In summary, the application principle of wet paper encoding in the model watermark is as follows: set the depth learning model parameter to a binary form $X={\left\{0,1\right\}}^{n}$; the added watermark information is $m={\left\{0,1\right\}}^{q}$ and *Y* is a hidden carrier. Any *k* element therein is used as the selection channel, and the sender is denoted as follows:

(2) Hv=m−DX

where *D* is a random ${\left\{0,1\right\}}_{q\times n}$ and *H* is a matrix composed of columns corresponding to *k* elements in *D*. After *v* is solved, the element corresponding to the nonzero element of *X* is modified to obtain a hidden carrier *Y*. The receiver can extract *m*, where m=DY.

**Figure 3:**
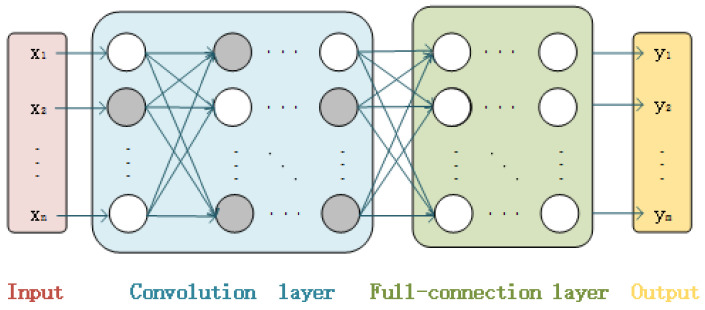
Schematic diagram of the application of WPC in the proposed scheme.

Figure 3 is a schematic diagram of the application of WPC in the scheme proposed in this paper. The gray neurons represent the unmodifiable important neurons selected by OPSS as wet blocks, and their position information is defined as the wet-block indicator in the WPC algorithm. The remaining white neurons are dry blocks that can be modified.

## 3. Proposed Scheme

In this section, we show that the proposed scheme is suitable for most deep learning models; the VGG19 model is used here as a specific example. Selecting the convolutional layers in the VGG19 model for steganography, a watermark can be added to the model without affecting the original function of the model by following the new proposed scheme combining OPSS and WPC, introduced in detail below.

First, the composition of the neuron parameters must be analyzed. A neural network is composed of many neurons, and each neuron is composed of many parameters. In the common PyTorch and TensorFlow frameworks, the parameters are all 32-bit floating point numbers. When the information is embedded, the parameters need to be converted from the decimal space to the nonnegative integer space. Specifying the neural network parameter as θ, the neural network parameters are set as θ. The floating-point number is transformed into a non-negative integer by I(θ) as follows:

(3) $$I\left(\theta \right)=\left(\theta +T\right)\cdot 10^{R}$$

where *T* is a translation that satisfies T>|min(Θ)|, θ+T translates negative numbers to the nonnegative space, and *R* is precision and indicates the number of decimal places retained. Then, θ+T can be converted to an integer by multiplying $10^{R}$. Obviously, the larger the value of *R*, the more decimal places are retained for the parameter, and the more accurate the recovered result is.

Nonnegative integers can be converted to floating-point numbers using Equation (4)

(4) $$I^{-1}\left(s\right)=s\cdot 10^{-R}-T$$

### 3.1. OPSS

Next, based on the PSS in Section 2.1, we propose an OPSS to obtain an improved scheme. We perform two-step operations in the OPSS. First, we calculates the expected parameter importance of the current entire layer and select the unimportant layer for the next step. The specific selection operation process is as follows. First, on the basis of Equation (1) the importance parameter $p_{\theta }$ of the parameter θ of each neuron in the *l*-th layer of $\theta^{l}$ can be obtained, then the importance layer parameter $L_{p_{\theta }}$ of the *l*-th layer can be calculated; that is, through the importance parameter $p_{\theta }$ of each parameter θ of the layer, the expected value of the layer is obtained by accumulating the importance parameters of all neurons in the current layer. As the value of $L_{p_{\theta }}$ should be between [0−1], the volume can be calculated. The unimportance layer formula for the buildup parameters is as follows:

(5) $${\hat{L}}_{p_{\theta }}=1-\frac{1}{n}\sum_{i=1}^{n}{p_{\theta }}^{i}$$

where *n* is the number of neuron parameters θ in the *l*-th layer and ${\hat{L}}_{p_{\theta }}$ is the unimportance value.

The unimportant layer value of each layer can be obtained through Equation (5). The top 80% of the unimportant layers can be selected through multiple tests in the fourth part, information is not embedded into the remaining important layers, and the original parameter information is retained, which can ensure that the original function of the model is not affected. c∈[0−0.8] is the ratio of the number of layers to be embedded with information to the total number; the larger *c* is, the more information can be embedded in the model during steganography.

After selecting the top-ranked unimportant layers, the second selection is based on the need to embed watermarks or the information size, then the relatively important parameters in the unimportant layers are selected to avoid the unimportant layers that have high $p_{\theta }$ values. The highly important parameters are selected as the wet block indicators of WPC in the watermark embedding process. These indicators ensure that the model can expand the amount of embedded information as much as possible under the condition of high fidelity.

**Figure 4:**
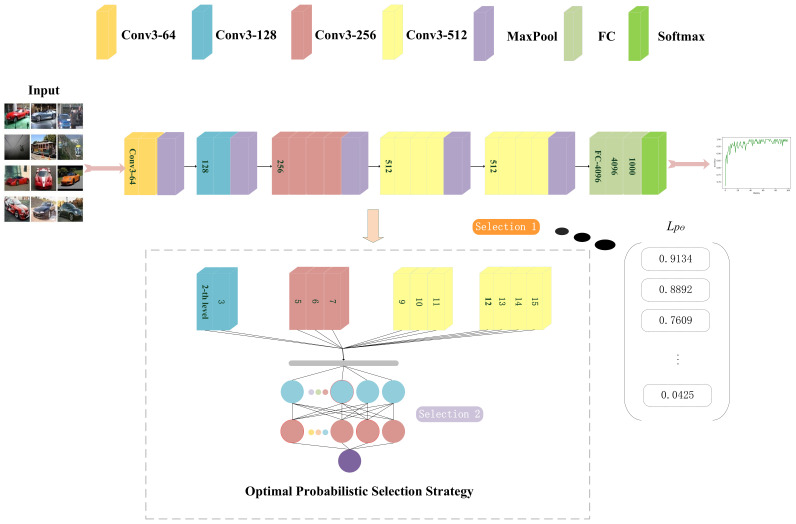
Schematic diagram of OPSS.

Figure 4 is a schematic diagram of the application of WPC in the scheme proposed in this paper. The gray neurons represent the unmodifiable important neurons selected by OPSS as wet blocks, and their position information is defined as the wet block indicators in the WPC algorithm. The remaining white neurons are dry blocks that can be modified. During Selection 1, we chose CIFAR-10 as the input data for the model. We calculate ${\hat{L}}_{p_{\theta }}$ (the value of the unimportance of the *l*-th layer) as a one-dimensional sequence using Equation (5) and sort this sequence. Then, we set the proportion *c* of the unimportant layer to 0.75 and obtain the result, which states that 0, 1, 4, and 8 are important layers and the rest are unimportant layers. Finally, we proceed to Selection 2 to select the relatively important parameters of the unimportant layers.

### 3.2. Our Watermark Embedding Algorithm

In this section, we show the algorithm for embedding watermarks in the proposed scheme. The process of watermark extraction is directly based on the process of secret extraction in Section 2.2:

Regarding Algorithm 1, we make the following comments.

1. The method for calculating the important parameters of the neural network model is not the focus of Section 2.1, therefore the important parameters of F are directly used as the input of the algorithm.
2. Wet-indicate represents the wet-block position, which denotes the position where the information cannot be embedded; the implicit information indicates that the embedded position is in these layers.
3. Watermark extraction. This is the inverse process of embedding watermarks; wet–indicate is obtained according to OPSS, *H*, *D*, and *k* are from Algorithm 1, and the watermark can be extracted using Equation (2).

**Algorithm 1:** The embedded watermark model.
**Input**: The model F (the model level l∈(1,2,…,L)), important parameters $\hat{\Theta }$ of F, value of important parameters $p_{\theta }$, neuron number set $N=[n_{1},n_{2},\ldots ,n_{l}]$, ratio of the number of layers to be embedded *c*, watermark information *B*, translation *T*, precision *R*, random matrix *D*, random value *k*, watermark *m* in a level.
**Output**: Watermarked model $F_{watermark}$.
**Step 1:** For each level to calculate the unimportance ${\hat{L}}_{p_{\theta }}=1-\frac{1}{n}\sum_{i=1}^{n}{p_{\theta }}^{i}$
**Step 2:** Sort ${\hat{L}}_{p_{\theta }}$, select the top c×L layers (a total number of *L* layers) in the order.
**Step 3:** Estimate the proportion of important parameters θ in these selected layers $\hat{L}$ by watermark information *B*; obtain wet–indicate, which represents the wet-block position.
**Step 4:** Convert floating-point θ into a nonnegative integer *b* (watermark information *B*) through $b=I\left(\theta \right)=\left(\theta +T\right)\cdot 10^{R}$. Then convert nonnegative integer *b* to floating-point $\theta^{\prime }$ through $\theta^{\prime }=I^{-1}\left(b\right)=b\cdot 10^{-R}-T$.
**Step 5:** Generate a random matrix *D* and a random value *k* and obtain *H* by *D* and *k*. Record these values.
**Step 6:***b* is divided equally, $\left[b_{1},b_{2},\ldots ,b_{m}\right]$ are denoted as *m*, *X* is obtained by wet–indicate in F.
**Step 7:** Embed watermark *m*; after embedding *m* each time, b=b−m. Repeat Steps 5–7 until b=0.


## 4. Experiments and Comparison

In this section, we report the results of experiments and analyses conducted to evaluate the fidelity, robustness, and embedding capacity of the proposed scheme in comparison with other model watermarking schemes.

Here, we briefly introduce the experimental setup, using a CNN model, the classification VGG19 model, to validate the proposed scheme. The proposed model is mainly used to recognize and classify the dataset images. After determining the training dataset, the model selects the hyperparameter settings and training strategies for different datasets. The hyperparameter settings and training strategies are listed in Table 2. We chose to embed a 2560-bit watermark (64-bit watermarks are already able to prove ownership of models, and a 256-bit watermark is sufficient for security in law). Our hardware platform was an Nvidia GTX 1080ti with an Intel(R) Core(TM) i7-10700F CPU@2.90 GHz and 64 GB of memory.

The datasets selected for the experiment were MNIST, CIFAR-10, and ImageNet. MNIST is a set of handwritten digits. The dataset contains 60,000 examples for training and 10,000 examples for testing. These numbers have been standardized in size and are located in the center of an image which has a fixed size of 28×28. CIFAR-10 is a small dataset for identifying universal objects. It contains RGB color pictures of ten categories: aircraft, automobile, bird, cat, deer, dog, frog, horse, ship and truck. The size of the picture is 32×32. There are 50,000 training pictures and 10,000 test pictures in the dataset. ImageNet contains 14,197,122 pictures and 21,841 classes, of which the commonly used subset includes 1000 classes and 1.2 million pictures. It is a widely used dataset in deep learning, with most current research work on image classification, location, and detection being based on ImageNet.

### 4.1. Fidelity Evaluation

We conducted 10,000 experiments on each dataset and used the average results to obtain the accuracy of the model before and after embedding the watermark. In Table 3, the fidelity evaluation of the three sets is listed. The original accuracy of the model is referred to as the baseline accuracy, while the accuracy of the watermarked model is the watermarked accuracy.

**Figure 5:**
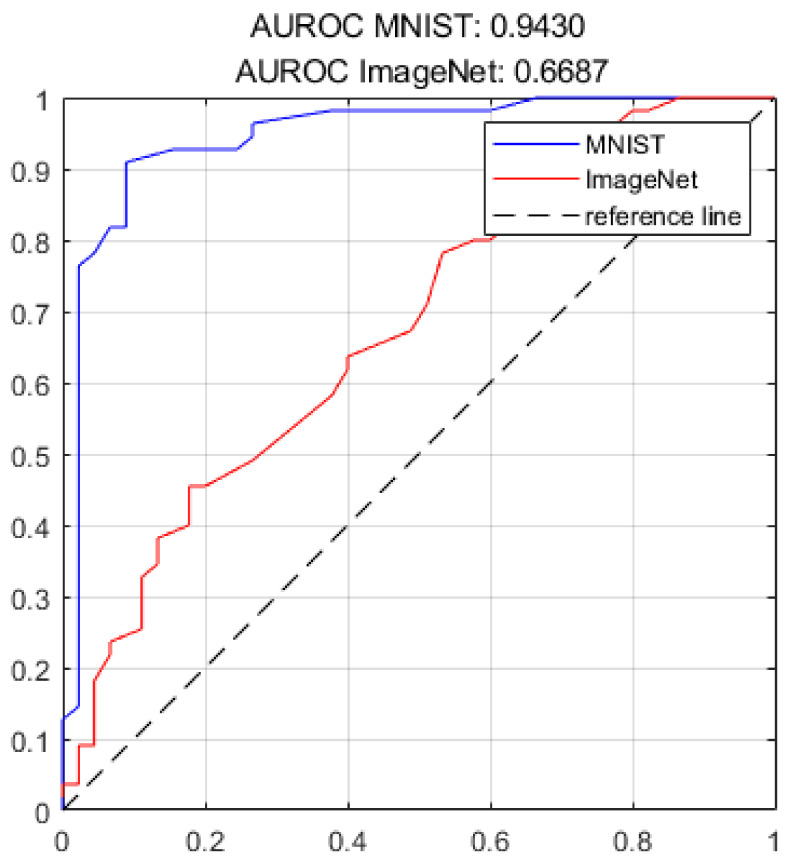
Area under the ROC curve of the watermarked model.

To fully prove the fidelity of the watermarked model, we used several common DNN performance metrics for MNIST and ImageNet The results are shown in Table 4 and Figure 5. The precision, recall, F1, and area under the curve (AUC) scores are used; these values depend on the true positive (TP), false negative (FN), true negative (TN), and false positive (FP) values. The corresponding descriptions of the performance metrics are shown below.

(6) $$Accuracy=\frac{TP+TN}{TP+FN+TN+FP}$$

(7) $$Precision=\frac{TP}{TP+FP}$$

(8) $$Recall=\frac{TP}{TP+FN}$$

(9) $$F1score=\frac{2TP}{2TP+FN+FP}$$

AUC score: The AUC statistic is an empirical measure of classification performance based on the area under a receiver operating characteristic (ROC) curve. The ROC curve plots sensitivity (TP rate) against specificity (FP rate), and AUC represents the degree of separability.

### 4.2. Robustness Evaluation

To evaluate the robustness of this model, we used several attack methods, which are introduced in detail below.

#### 4.2.1. Resisting Fine-Tuning

In most transfer learning scenarios, users need to fine-tune the model using their own datasets. Therefore, for a watermarked model the watermark should not be destroyed by a user’s fine-tuning of the model.

**Figure 6:**
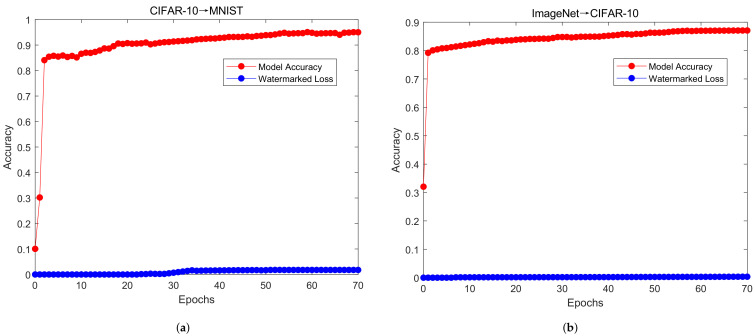
Changes in model accuracy and watermark loss in watermarked models as the number of fine-tuning epochs increases.

We set up two sets of experiments; CIFAR-10 was used to fine-tune the watermarked MNIST model, and ImageNet was used to fine-tune the watermarked CIFAR-10 model. Due to the differences in the datasets, we made the following adjustments. First, we adjusted the size of the images in CIFAR-10 to be the same as those in MNIST. Next, as the output layer of ImageNet includes 1000 neurons, when fine-tuning it with CIFAR-10 the output layer needed to be modified to include ten neurons. In order to fully consider the robustness of the watermark, we assume that all layers of the model are fine-tuned and the training strategy for fine-tuning follows the settings in Table 2. As shown in Figure 6, we obtain the accuracy results of the fine-tuning process. Furthermore, we can observe that, in the left figure, when the number of epochs is approximately 30 the accuracy of the model can reach more than 90%. The accuracy of the model can reach nearly 95% when the CIFAR-10 dataset is used to fine-tune the MNIST model as well. On the right, when the number of epochs is approximately 50, the accuracy of the model can reach more than 85%, and the accuracy of the model can reach nearly 90% when using the ImageNet dataset to fine-tune the CIFAR-10 model. Even if the model is fine-tuned, the watermark is not destroyed.

At the beginning of training, the accuracy rate rises rapidly and the training loss decreases significantly, indicating that the learning rate is appropriate and the gradient decline process is carried out. After learning to a certain stage, the accuracy rate and loss curve of the model tend to be stable and slowly reach a certain fixed value. The problem of test loss is expressed here as the characteristics of different datasets affecting the training model. In the process of fine-tuning, the loss of watermark is another expression of test loss. We can easily find that the watermark loss is almost 0 and test loss is minimal for the proposed model.

#### 4.2.2. Resisting Watermark Overwriting

In our watermark scheme, a watermark is subtly embedded in the weights of certain layers via OPSS. When an attacker uses a watermark overwriting attack, because they do not know where the watermark is embedded, it is difficult for them to destroy the original watermark of the owner. Obviously, small capacity coverage cannot affect our watermark model; therefore, we chose thousands of levels of watermark coverage, as shown in Table 5. Even if an attacker overwrites a 1024-bit or 2560-bit watermark in the model, the loss of the original watermark is almost negligible. Of course, an attacker can try to overwrite more watermarks; however, as more watermarks that are embedded, the accuracy loss of the model increases, eventually making the model unusable.

We added an ablation experiment focusing on the capacity of the embedded watermark, which ranges from 512 bits to 5120 bits. The change in accuracy of the model is shown in Table 6. When the amount of embedded information changes, the resulting change in model performance can be observed. According to the results, we find that with increased information embedding the accuracy of the model is not greatly affected. The reason is that the proposed OPSS in this paper ensures that the important parameters of both the important and unimportant layers in the deep learning model are not embedded; thus, the active neurons work normally. As the prediction results of the model mainly depend on these neurons, the accuracy of the model changes only slightly. This further verifies that the model has a large embedding capacity.

### 4.3. Embedding Capacity

The embedded watermark is a highlight of our scheme. Notably, while there are a total of approximately $2.0\times 10^{7}$ convolution layer neuron parameters in VGG19, the main parameters of the model only account for a few, i.e., less than 8%, of the total model parameters. Furthermore, the embedded information is related to the input data.

We set c=0.75, as shown in Table 7. Even if we modify the neuron parameters in only 75% of the convolutional layers, the results at this information embedding rate are as high as 71.98%.

### 4.4. Comparison with Previous Schemes

In order to fully demonstrate the superiority of our proposed scheme, in this section we compare it to several previous works.

All comparison results are listed in Table 8, and the results are analyzed in detail below. In the course of our comparison we found that while the different proposed schemes all have favorable performance, they have different drawbacks. The main drawback of Uchida’s scheme is that it cannot resist watermark overwriting attacks. The disadvantage of Rouhani’s scheme lies in its lower watermark capacity, with obvious watermark loss occuring when a 128-bit watermark is embedded. Although Feng and Zhang’s scheme has a high embedding capacity, we believe that there is considerable watermark information embedded in the model in this scheme, which is not a sensible approach. Fan’s scheme requires changing the structure of the model; while it can prevent an attacker from embedding a new watermark, this approach consumes a very large amount of time. To ensure that the passport layer is not detected by the user, this approach takes more than twice as long to train, which greatly limits its use in real scenarios. Researchers at Tencent Suzaku Lab proposed a method of using LSB image implications in which they directly selected the neural parameters to be changed. However, when this method restores the watermark it is necessary to record the location information of a large number of changed parameters. The disadvantage of Wang’s scheme is that the parameters are directly replaced according to the entire layer, resulting in the embedded information being easily detected and not being random or secure. This approach cannot resist certain types of model attacks, and the watermark is easy to find and destroy. We believe that our scheme makes up for the drawbacks of these schemes. The specifics have been described in the experimental evaluation, and are not repeated here. The embedding cost refers mainly to the time required to calculate the importance of the model parameters; the scheme has a low cost and can ensure the safety, high fidelity, strong robustness, and very high embedding rate of the model.

## 5. Conclusions

This paper proposes the use of an OPSS model to select the important parameters of the original DNN. The positional information of the important parameters is then recorded as a wet-block indicator in the WPC and watermark information is embedded in the position of the unimportant parameters, ensuring that the model has high fidelity. Compared with previous schemes, our scheme can prevent malicious tampering by attackers and has a higher information embedding rate. The proposed model performs brilliantly in various tests. For example, it performs well in terms of model fidelity, robustness, and capacity, and shows both reliability and practical application significance. The large-capacity availability of the deep learning network watermarking model proposed in this paper is worth considering. Instead of embedding watermarks, the model can embed other meaningful or valuable information to achieve different purposes; for example, it can embed malicious code as an attack method or carry information for secret sharing. We intend to continue to explore this topic and discover new ways to evolve neural network watermarking.

## Figures and Tables

**Table 1:** The information embedding ratio is the ratio of all parameters of the model to the amount of information embedded in the model.

Parameters	Definition
$F_{\Theta }$	The DNN for watermark embedding
$\Theta^{l}$	The parameters of the *l*-th layer
$\hat{\Theta^{l}}$	The selected important parameters
$p_{\theta }$	The importance value of each parameter θ
$L_{p_{\theta }}$	The importance layer parameter
${\hat{L}}_{p_{\theta }}$	The unimportance layer parameter
*N*	A collection of the number of neurons in each layer, expressed as $\left[n_{1},n_{2},\ldots ,n_{l}\right]$
*n*	The number of neural parameters in a level
*c*	The ratio of the number of layers to be embedded
*B*	Watermark information
*X*	The carrier is ${\left[0,1\right]}^{n}$
*m*	The secret is ${\left[0,1\right]}^{q}$
*k*	The number of neurons embedded in a layer
*D*	A random matrix is ${\left[0,1\right]}^{q\times n}$
*H*	A matrix consisting of columns corresponding to *k* elements in *D*
wet–indicate	The location address of a wet block

**Table 2:** Hyperparameter settings of VGG19.

Dataset	Batch Size	Epochs	Optimizer	Learning Rate	Learning Rate Update Strategy
MNIST	64	30	Adm	0.001	Learning rate is fixed
CIFAR-10	64	70	Adm	0.001	Decrease by 1/10 each 30 epochs
ImageNet	64	70	Adm	0.001	Decrease by 1/10 each 30 epochs

**Table 3:** Fidelity evaluation of three sets of watermarked models.

Dataset	Baseline Accuracy	Watermarked Accuracy
MNIST	95.68%	95.73%
CIFAR-10	92.36%	92.29%
ImageNet	68.84%	68.26%

**Table 4:** Fidelity evaluation of two sets of watermarked model.

Dataset	Precision	Recall	F1Score
MNIST	95.32%	94.66%	94.18%
ImageNet	66.70%	68.46%	67.88%

**Table 5:** Reserve of the original watermark after overwriting a 1024-bit or 2560-bit watermark.

Dataset	Overwriting Capacity-1024 bits	Overwriting Capacity-2560 bits
MNIST	99.08%	98.23%
CIFAR-10	98.74%	98.16%
ImageNet	97.39%	96.58%

**Table 6:** The relationship between the capacity of the embedded watermark and model accuracy using MNIST, CIFAR-10, and ImageNet.

Dataset	Baseline Accuracy	Capacity (bits)	Watermarked Accuracy	Fine-Tuning Accuracy (30 Epochs)
MNIST	95.68%	512	95.68%	95.65%
	95.68%	1024	95.71%	95.68%
	95.68%	2560	95.73%	95.60%
	95.68%	5120	95.80%	95.73%
CIFAR-10	92.36%	512	92.36%	92.39%
	92.36%	1024	92.37%	92.38%
	92.36%	2560	92.29%	92.35%
	92.36%	5120	92.40%	92.41%
ImageNet	67.84%	512	67.82%	67.89%
	67.84%	1024	67.60%	67.74%
	67.84%	2560	67.26%	67.37%
	67.84%	5120	66.89%	67.11%

**Table 7:** The information embedding rate is the rate of all parameters of the model and the amount of information embedded in the model.

Dataset	Information Embedding Rate
MNIST	71.98%
CIFAR-10	70.04%
ImageNet	66.35%

**Table 8:** Comparison of different algorithms (N means failed, Y means passed).

Algorithm	Fidelity	Robustness	Capacity
Uchida	Y	N	High
Rouhani	Y	Y	Low
Fan	Y	Y	-
Feng and Zhang	Y	Y	High
Researchers of Tencent Suzaku Lab	Y	Y	High
Wang	Y	N	High
Ours	Y	Y	Very High
